# Feasibility, Safety, and Compliance in a Randomized Controlled Trial of Physical Therapy for Parkinson's Disease

**DOI:** 10.1155/2012/795294

**Published:** 2011-12-10

**Authors:** Jennifer L. McGinley, Clarissa Martin, Frances E. Huxham, Hylton B. Menz, Mary Danoudis, Anna T. Murphy, Jennifer J. Watts, Robert Iansek, Meg E. Morris

**Affiliations:** ^1^Melbourne School of Health Sciences, Physiotherapy, The University of Melbourne, Carlton, VIC 3010, Australia; ^2^National Parkinson Foundation Center of Excellence, Clinical Research Centre for Movement Disorders and Gait and Victorian Comprehensive Parkinson's Program, Kingston Centre, Warrigal Road, Cheltenham, VIC 3192, Australia; ^3^Musculoskeletal Research Centre, Faculty of Health Sciences, La Trobe University, Bundoora, VIC 3086, Australia; ^4^Centre for Health Economics, Monash University, Building 75, Clayton, VIC 3800, Melbourne, Australia

## Abstract

Both efficacy and clinical feasibility deserve consideration in translation of research outcomes. This study evaluated the feasibility of rehabilitation programs within the context of a large randomized controlled trial of physical therapy. Ambulant participants with Parkinson's disease (PD) (*n* = 210) were randomized into three groups: (1) progressive strength training (PST); (2) movement strategy training (MST); or (3) control (“life skills”). PST and MST included fall prevention education. Feasibility was evaluated in terms of safety, retention, adherence, and compliance measures. Time to first fall during the intervention phase did not differ across groups, and adverse effects were minimal. Retention was high; only eight participants withdrew during or after the intervention phase. Strong adherence (attendance >80%) did not differ between groups (*P* = .435). Compliance in the therapy groups was high. All three programs proved feasible, suggesting they may be safely implemented for people with PD in community-based clinical practice.

## 1. Introduction

Physical rehabilitation in Parkinson's disease (PD) is a growing field of investigation. Although a number of small randomized controlled trials (RCT) have reported some benefits of physical rehabilitation programs for people with PD [1–5], the outcomes of recent systematic reviews remain equivocal [6–8]. There are many excellent examples of clinical trials, particularly those evaluating rehabilitation outcomes (e.g., [2, 4, 9]). The existence of such rich literature highlights the importance of ensuring high levels of adherence and compliance with therapy protocols, as well as carefully tracking attrition and adverse responses [10]. This manuscript addresses that gap.

The conduct of clinical research presents challenges; trial outcomes can be influenced by many variables related to the rigor of research methods employed, such that even carefully planned and well-funded clinical trials can fail to yield high-quality data or allow results to be translated into practice. In addition, exercise modalities aimed at strengthening and preventing falls may present safety risks to the potentially frail and debilitated participants enrolled in physical rehabilitation clinical trials. 

Participant retention has been identified as an issue in a number of previous randomized controlled trials of physical rehabilitation in PD, particularly those studies involving an inactive control group [11, 12]. It has been suggested that offering an alternative therapy as a control intervention may improve retention in nonpharmacological randomized clinical trials [11]. Without the option of blinding participants to group allocation within a physical rehabilitation clinical trial, the challenge becomes developing alternative therapy programs that offer participants an equivalent participation experience whilst minimizing overlap with the content of the active intervention.

Adherence and compliance are key variables influencing the outcome of clinical trials in PD [13]. Within the context of a physical rehabilitation trial, it is important to establish not only when participants attend (adherence; [1, 13]), but what activities they complete during their attendances, that is, the extent of their engagement with the program (compliance; [13]). Adherence and compliance, therefore, reflect the adequacy and appropriateness of therapy content for the sample and should be considered within the design of a clinical trial by the development of appropriate therapy and training protocols. 

To date, few trials of physical rehabilitation programs for PD have reported adherence and compliance data (e.g., [14]). Fewer studies have described in detail the strategies used or recommended to maximize adherence and compliance in this patient group. The purpose of this paper is to report the safety, retention, adherence, and compliance rates of a large RCT investigating the efficacy of physical rehabilitation to reduce falls and improve mobility in people with PD. In addition, strategies to improve adherence and compliance will be described, and implications for future research will be discussed.

## 2. Methods

### 2.1. Study Design

We conducted a single blind randomized controlled trial to evaluate the effectiveness of two methods of physical therapy combined with falls education to improve mobility and decrease falls in people with PD, relative to a control intervention [15]. Ethical approval was gained from the relevant Ethics Committees, and all participants provided written informed consent.

### 2.2. Participants

A convenience sample of 210 participants with idiopathic PD was recruited between 2006 and 2009 throughout Melbourne, Australia from neurologists and therapists working in clinics and rehabilitation centers, from PD support groups and from community newspaper advertisements. Eligible people were those who: (i) had a confirmed diagnosis of idiopathic PD; (ii) were able to walk (Hoehn and Yahr (HY) Stages 0-IV [16]); (iii) had a Mini Mental State Examination (MMSE) score ≥24; (iv) were willing and able to attend the therapy and assessment program. Exclusion criteria were other medical conditions that could limit or prevent exercising safely at the required intensity, other prior neurological conditions affecting gait, and dementia.

After screening and consent, participants were randomized to one of three groups: progressive strength training (PST) combined with falls prevention education; movement strategy training (MST) combined with falls prevention education; or a control group (life skills; LS).

### 2.3. Intervention

The programs were delivered by clinical staff employed in outpatient settings. All staff delivering the intervention completed 2.5 hours training on the therapy protocols, conducted by the study chief investigators. The interventions were delivered in a once weekly two-hour session for 8 weeks to groups of 3-4 participants. The PST and MST interventions were delivered by a physical therapist, and the LS program was delivered by occupational therapists or social workers. An allied health assistant also attended sessions as required to provide general assistance. In addition, all participants were provided with a home exercise program to be completed independently or with carer/family assistance once a week. 

The interventions are described in detail elsewhere [15, 17]. To summarize, the PST program comprised seven strengthening exercises for core muscle groups of the lower limbs and trunk, in accordance with the principles of PST [18]. Exercises were progressed by adjusting the number of sets and repetitions, by adding more weights to the vest, by increasing the Thera-band (stretch elastic band) resistance and by adjusting the step or chair height. Exercises were individually tailored and progressed, taking into account factors such as age, fitness level, comorbid health conditions such as arthritis or back pain, and self-reported exercise difficulty according to the Borg Perceived Exertion Scale [19]. The individualized home exercise program was recorded on a standardized home exercise sheet template each week by the therapist. A booklet with photos of each exercise, a gym step, vest with weights, and Thera-band were supplied for use at home during the intervention phase.

The MST program comprised the individualized teaching of training strategies to enhance movement performance, improve balance and mobility, and to prevent falls, according to the principles outlined by Morris et al. [20–22]. Participants practised using strategies such as attention, verbal, and external cues while performing seven functional tasks such as sit to stand, moving from chair to chair, standing and reaching, or walking and turning, either in single or dual task conditions. A booklet with photos and details of each exercise was provided to each participant. Exercises were individually tailored to the functional level of each participant, and progression of each task varied according to need and ability. A home exercise program tailored to the individual's level was prescribed each week by the therapist.

Both the PST and MST groups received 10–15 minutes of structured falls education component at each weekly session, incorporating an overview of risk factors and strategies to prevent falls. This was based upon the content of the booklet: *Don't fall for it. Falls can be prevented!—A Guide to Preventing Falls for Older People* booklet [23].

The control intervention comprised guided discussion sessions on PD-related topics such as the impact of PD on the individual and family, fatigue management, relaxation, medication, communication, and community services. The LS session did not include any content related to falls education, exercise, walking, or balance. Therapists also suggested activities such as reflection activities and relaxation practice to be completed once a week at home.

### 2.4. Outcome Measures

All participants were tested by trained blinded physical therapist assessors at baseline (T1), one week after the 8-week intervention (T2), and at 3 months (T3) and 12 months (T4) after intervention. The primary outcome measure was falls over 12 months after-intervention, as detailed previously [15]. Secondary outcome measures included measures of mobility, activity limitations, and quality of life.

### 2.5. Outcome Measures for the Intervention Phase

Intervention therapists recorded key details of therapy delivered after each session using custom designed forms. The therapy record for the intervention groups indicated compliance with key therapy concepts. For the MST group, this reflected the individual tailoring of activities to address functional movement difficulties. For the PST group, the record detailed the exercises, number of repetitions and sets, weights, and Thera-band resistance level. All participants were screened weekly at the intervention sessions for new adverse events, including new muscle soreness related to therapy. Falls were monitored using a Falls Calendar protocol [15]. This required people to enter falls on a calendar as they occurred and to telephone a falls hotline to answer questions relating to fall circumstances and consequences. Falls Calendars were completed during the intervention phase and for 12 months following intervention. 

For the purposes of this study, feasibility was adopted as an umbrella term, encompassing the constructs of safety, retention, adherence, and compliance. *Safety* during the intervention phase was monitored by: (i) structured weekly screening by the intervention therapists for any new soreness lasting longer than 48 hours related to therapy; (ii) recording of adverse events that occurred during therapy; and (iii) fall rate during the intervention phase. *Retention *was defined in several ways: (i) the proportion of participants who attended the first post-intervention assessment; (ii) the proportion of participants who returned post-intervention Falls Calendars (reflecting the primary outcome measure of the overall trial); (iii) the proportion of participants who completed all follow-up assessments compared to the number who completed baseline assessments (note that this measure is the opposite of “dropouts”). *Adherence* considered the consistency of participant attendance at the intervention/control sessions. *Compliance *to the intervention was determined by the progression of exercises within each of the two intervention groups as evidenced by therapy records.

### 2.6. Data Analysis

Demographic data were gathered for each group for variables such as age, sex, disease duration, past history of falls, and comorbidities. Kaplan-Meier survival analysis was used to examine time to first fall during the intervention phase of the trial and compared between groups using Mantel-Cox log rank test. Between-group comparisons of adherence were assessed using an independent samples one-way Kruskal-Wallis test. Data were analysed using IBM SPSS version 19.0 (SPSS Corp, Chicago, Ill, USA) or STATA 8 (Stata Corp, College Station, Tex., USA) statistical software.

## 3. Results

### 3.1. Participants

Two hundred ten participants (140 men, mean age (SD) of 67.9 (9.6), range 44–89 years) were randomized. Participants generally had mild to moderately severe PD, reflected by a median modified HY stage (IQR) of 2.5 (2-3) and mean (SD) disease duration of 6.7 (5.6) years. Activity limitations, as measured by the Unified Parkinson's Disease Rating Scale (UPDRS) Part II activities of daily living, were also mild (mean (SD); 11.6 (5.9)). One hundred sixteen participants (55%) reported having falls over the previous 12 months, of whom 74 (64%) were repeat fallers. Freezing of gait was reported by more than half of the participants. Arthritis was the most commonly reported health condition, present in 92 (44%) of the sample, and 48 (23%) participants had a history of cancer or heart disease. The majority of participants were taking levodopa preparations or a combination of PD medications, with 19 on no PD-pharmacotherapy. One hundred fourteen (54%) participants were prescribed four or more medications, with 89 (42%) taking psychotropic medication.

### 3.2. Delivery of Interventions

The interventions were undertaken in four different outpatient centers located in different regions of Melbourne. Across the three years of the RCT, 8 physical therapists delivered the MST, 10 physical therapists delivered the PST and 6 occupational therapists or social workers delivered the LS program. Therapist professional experience varied markedly from new graduate (<1 year) to highly experienced (>30 years).

### 3.3. Safety

The safety of the interventions was assessed in three ways and is reported in Table 1. Structured weekly screening during the intervention phase identified new soreness lasting longer than 48 hours in 28 individuals (PST *n* = 18; MST *n* = 10). Seven individuals reported more than one episode of soreness (PST *n* = 6; MST *n* = 1). Typical reports included a transient increase of preexisting low back, hip or knee pain related to osteoarthritis, resolved by a modified program or over-the-counter medication. Fewer than one quarter of these participants attended a health service practitioner because of new soreness. No new soreness was reported to persist beyond the intervention phase and require intervention. 

Secondly, three incidents occurred during the actual intervention sessions. Two MST participants reported single episodes of dizziness with subsequent medical assessment that were resolved without intervention or sequelae. A single participant from the PST group fell during the therapy session, with no reported injury. None of these incidents resulted in any ongoing consequence.

The third safety evaluation examined falls in 203 participants during the intervention phase. Fifty-eight people fell during this phase: (PST *n* = 10, MST *n* = 24, LS *n* = 24). Falls frequency varied markedly; 32 people fell once or twice; 19 fell between 3 to 9 times; 7 fell 10 or more times. The median time to the first fall during the intervention phase was 14 days in the PST group and 9 days in the MST and LS groups. The time to first fall did not differ significantly between groups; Log rank test (Mantel Cox), Chi square = 2.08, df = 2, *P* = 0.353.

### 3.4. Retention

Three aspects of retention of participants were considered related to attendance at the three posttherapy assessments and the return of Falls Calendars. The study protocol had allowed for a drop-out rate of 15% when determining the required sample size. Seven participants, six in the LS group and one in the MST group, withdrew prior to the intervention phase after being randomized to a group. Reasons for withdrawal included poor health (LS *n* = 2), a preference for the exercise group (LS *n* = 1), unable or no longer wanting to attend (LS *n* = 2, MST *n* = 1), and deceased (LS *n* = 1). Eight participants withdrew from the study during or after the intervention phase and did not return Falls Calendars during the 12 months follow-up phase. Six of these withdrew from the LS program, two due to health reasons, one as they did not want to continue (unspecified reason), one because he felt the group was “depressing”, and two as they were not exercising or receiving falls education. One participant withdrew from PST for health reasons, and one participant from the MST group died of unrelated causes. One hundred ninety-six (93%) of the participants completed the T2 assessment at the end of the 8-week intervention phase (PST *n* = 69; MST *n* = 68; LS/control *n* = 59; see Table 2). 

Retention throughout the full trial period was high. One hundred ninety-five participants (93%) returned one or more Falls Calendars during the 12-month follow-up period (PST *n* = 69, MST *n* = 67, LS *n* = 59). One hundred eighty-four participants (88%) provided falls data for the entire 12 months (PST *n* = 65, MST *n* = 65, LS *n* = 54). In the final evaluation of retention, 775 assessments of possible 840 (210 × 4 occasions) were completed (92%). Participation at the final T4 assessment as a percentage of the total number randomized showed 93% of people in the PST group were reassessed, 91% of MST and 79% of participants in the LS program.

### 3.5. Adherence

Eight participants were randomized, but did not attend any therapy sessions (PST *n* = 0, MST *n* = 2, LS *n* = 6). Adherence data are reported for the participants who attended at least one intervention session (PST *n* = 70, MST *n* = 67, LS *n* = 65). Ninety percent of the PST participants attended between 6 and 8 sessions, with 3 participants (4%) attending fewer than 5 sessions. Ninety-three percent of the MST participants attended 6–8 sessions, with 2 participants (3%) attending fewer than 5 sessions. Seventy-eight percent of the LS participants attended between 6 and 8 sessions, with six participants (9%) attending fewer than 5 sessions. Participant attendance (as defined by attendance at ≥6 sessions or 75%) did not differ across the three groups (independent samples Kruskal-Wallis, *P* = .435). The PST group attended 82.5% of available sessions, the MST group 90.5%, and the LS group 80.7%.

### 3.6. Compliance

#### 3.6.1. Progressive Strength Training Group

A review of the therapy records indicated that 89% of the participants were able to complete all seven suggested exercises within the 2-hour session. The remaining 11% were able to complete six exercises. Increasing the number of repetitions and/or sets was the most common form of progression, with 97% of participants (68 of 70) progressing in this manner. Eighty percent (56 of 70) of the participants used the vest with weights during the appropriate exercises. Of these, only 5 participants (9%) did not increase the weights across the course of the intervention. Both the step platform and Thera-band (to resist trunk extension/rotation) were used by all participants. Thera-band resistance was increased for 57% of participants.

#### 3.6.2. Movement Strategy Training Group

A review of the available therapy records (*n* = 64, missing data = 3) indicated that over 86% (55/64) of the participants were able to routinely complete six or all seven activities within the 2-hour period. Increasing the number of repetitions and sets was the most common form of program progression, in conjunction with increasing the difficulty of the task. Progression of the task was highly variable according to each individual's task performance. For example, standing and reaching to an object in front of the participant may have progressed to moving the object further away, standing and placing an object down on the ground or up on a high shelf, to moving a heavier or more cumbersome object. Similarly, walking a straight line with long steps might have progressed to walking with a secondary motor task, with a secondary cognitive activity, to an obstacle course; standing up from a chair may have progressed by altering the height or compliance of the chair, or to standing up with an object in hand or standing up and walking off.

## 4. Discussion

The primary RCT described in this paper investigates the ability of two types of physical therapy to prevent falls in community-dwelling people with PD. The current secondary examination of feasibility demonstrates that these therapy programs can be successfully implemented within the context of an RCT. It also suggests they are feasible and may be safely translated to clinical practice.

### 4.1. Safety

As it was possible that these physical therapy programs might present safety risks, the first aspect of feasibility considered was the safety of the two active interventions. Both physical therapy interventions carried potential risks, either inherent in their content or specific to the population being treated. Each intervention aimed to extend participants to a high level of activity and performance, as high intensity exercise has been shown to be achievable in people with PD [24, 25]. The possibility, thus, existed of some consequent muscle soreness and/or joint stiffness, leading to the definition of a treatment-related minor adverse event as “soreness that lasted more than 48 hours or required attendance at a health professional.” Falls risk was potentially increased by aspects of the MST program that targeted and challenged aspects of motor performance such as balance, reach, and stride length. The PST program explicitly encouraged participants to work with increasing weights and resistance, potentially risking muscle, and joint problems. Participants were primarily older people (mean age 67.9 ± 9.7 years), potentially carrying a relatively high proportion of orthopedic conditions (osteoarthritis, osteoporosis) and other comorbidities [26]. Further, PD itself is strongly associated with impaired balance and increased falls risk [1, 27]. Finally, there was the possibility that increased confidence, as a result of intervention, might increase activity or risk taking and result in further falls. Evaluation of the safety of these interventions was therefore of key importance.

During the intervention, no adverse events with sequelae were reported. There were no injuries during the therapy sessions. Only 36 instances of “increased soreness > than 48 hours” occurred after the 1367 sessions attended, many in individuals with a history of back pain or osteoarthritis. A number of people were unsure whether it was the intervention or concurrent activities such as gardening or exercise that had triggered the soreness, and a visit to a health professional was necessary in fewer than one quarter of the instances reported. These results support the safety of the PST and MST programs in an older population with mild-moderate disability with a range of comorbidities. 

There were no group differences between the time to first fall during the intervention phase. This suggested that neither working to improve participants' functional motor performance in the MST group nor increasing their functional strength in the PST group may have led them to undertake risky behaviors and fall as a result of overconfidence. We conclude that both therapy programs can be safely implemented in this population.

#### 4.1.1. Retention

A key factor in achieving meaningful results from an RCT is the retention of adequate participant numbers. Retaining participants from any older population in a clinical trial over 12 months or more can be difficult [12, 13]. The retention level in the current trial exceeded expectations in all three measures relating to post-intervention assessment and Falls Calendar data. Falls data for the full twelve months were available from 184 (88%) participants. This compares very favorably with returns of 78% over 6 months in people with PD [27] and over 75% return of monthly falls questionnaires in elderly fallers [28].

The other measures of retention, attendance at T2 and attendance at all 4 assessments over baseline attendance (14 months from randomization), achieved greater than 90 percent retention, very similar to the 92% achieved over 6 months by Tickle-Degnan et al. [29]. Two other RCTs in PD reported differing retention rates over 12-months as measured by attendance at assessments. Only 51 percent of people with PD in one 12 month randomized controlled crossover trial attended all three of the assessments [12], whereas results equivalent to ours were found in a much smaller (*n* = 56) study of Qigong with almost 94 percent of people returning for their 12 months assessment [30]. 

Differential attrition between intervention and control groups can affect the equivalence of the groups achieved by randomization at the outset of a trial [31, 32]. In most cases, attrition tends to be greater in the control group [12, 30], although sometimes the intervention carries a level of adverse effects that may cause more people to drop out of the active group [33, 34]. Whilst more participants were lost to follow up in our control group than in the therapy groups, the differences were small. The provision of a control intervention that was similar in duration, group dynamics, and relevance to the exercise interventions appeared to optimize retention and may have limited dropouts.

#### 4.1.2. Adherence

Adherence, as defined by session attendance during the intervention phase, was also satisfactory with over 80% of available sessions attended by each group. Two smaller RCTs in PD (*n* = 68 and 116 resp.) reported adherences of 93% [2] and between 86% and 92% over 6 weeks intervention periods [2, 29]. Other reports of adherence in the literature are either over much longer periods [12, 14, 30], in different populations (e.g., [13, 28]), or involved physical therapy in the home setting [1, 9]. The determinants of satisfactory adherence rates are likely to be complex. There may be a degree of selection bias, as people who are willing to participate in research may be more likely to adhere to a program than those who refuse. Other factors such as locale, professional supervision by physical therapists, and social interaction may be relevant [35].

#### 4.1.3. Compliance

Evaluation of therapist and participant compliance to the protocol interventions is important to interpreting the key results of a trial. It also determines how effectively the interventions can be implemented as treatments in the wider context. Despite this, it is seldom reported. In our study, over 85% of participants were able to complete all or nearly all of the prescribed exercises, despite a mean age of 68 years and mild to moderate signs of PD (median modified HY of 2.5). Only one other paper, to our knowledge, reports the ability of people with PD to comply with the content of a therapy intervention to reduce falls [14]. In this small RCT (*n* = 48), compliance with 6 months of exercise therapy performed primarily at home was evaluated. Only 25% of participants were able to complete all prescribed exercises, with another 25% completing fewer than half of them, possibly because motivation may be different when exercising alone.

We believe compliance with therapy content was enhanced by the booklet of photographically illustrated exercise descriptions provided to each participant in the two exercise groups. Enlarged photographs were also placed at exercise stations in the various therapy locations to improve the accuracy of exercise performance, and correct performance was further facilitated by the presence of both a physical therapist and a trained assistant. The therapy protocol clearly directed that each participant should be working at a hard but achievable level (modified Perceived Exertion Scale levels [19]) that should have fully engaged participation. 

The strong compliance with content may also reflect the participants' relationship with and confidence in the treating therapists as well as the therapists' confidence in the trial exercise protocol. Importantly, the therapist was always the final judge of how the participant was to perform each activity and at what level, supporting their professional skill and understanding. As this level of compliance with program content was achieved by a number of different therapists of widely varying years of clinical experience, it appears that the content was well defined and easy to implement.

Although not formally assessed, many individuals from our three groups volunteered that they had enjoyed their participation. In part, this probably reflected the supportive relationships and camaraderie that developed between group members, reducing social isolation [36]. It was informally observed that group members supported each other despite differing levels of disability. Such information sharing has been reported as a desired outcome in PD [36]. These factors are likely to have enhanced adherence and compliance in the therapy groups. 

An important aspect to designing a randomized controlled trial is setting up the control group. Ideally, a control group should be exposed to similar duration and intensity of contact time as the intervention group, meeting the needs for education, attention, and socialization. The results of this study suggest that the LS program fulfilled these aims. The group's focus on PD specific topics [36] such as medication management, fatigue management, and communication was a strong point, building on the camaraderie and mutual support provided by members to each other. Control groups can often suffer from poor retention and adherence [12], particularly if they are simply a “wait list” group. Our results and others [11, 37] suggest that participant-relevant education helps improve group participation, particularly if social interaction and support within the group can be fostered. We conclude that guided small group discussions on topics of relevance can be recommended as a viable control program in the design of controlled clinical trials.

## 5. Conclusions

In the rehabilitation literature, there are few reports of the feasibility, safety, and adverse events associated with physical therapy for people living with Parkinson's disease. Our results address this gap and show that, when combined with a falls education program, strategy training and strength training can be safely implemented in a community-based sample of people with idiopathic PD. We also found that a life-skill social and education program was an effective control intervention that maintained interest without providing the active ingredients of therapy. Protocols could be easily followed by clinicians with varying levels of expertise, allowing for replication in future trials throughout the world.

## Figures and Tables

**Table 1:** Safety during the intervention phase.

	PST	MST	LS
“New soreness > 48 hrs.”			
Occasions of new soreness (*n* = 36)	25	11	0
Participants reporting new soreness (*n* = 28)	18	10	0

Incidents during therapy sessions	1 (fall; no sequelae)	2 (dizziness; no sequelae)	0

Falls during intervention phase			
Number of fallers (*n*)	10	24	24
Falls frequency: median (IQR)	0 (0)	0 (0-1)	0 (0-1)
Falls frequency: range (*n*)	0–7	0–24	0–52
Median time to first fall (days)	14	9	9

**Table 2:** Assessments attended across the course of the trial.

Assessment	PST *n* (% of randomized)	MST *n* (% of randomized)	LS *n* (% of randomized)
TI (Baseline assessment)	70 (100)	69 (100)	71 (100)
T2 (1 week after intervention phase)	69 (98.6)	68 (98.6)	59 (83.1)
T3 (3/months after intervention phase)	67 (95.7)	64 (92.8)	54 (76.1)
T4 (12 months after intervention phase)	65 (92.9)	63 (91.3)	56 (78.9)
